# Body Composition Relationship to Performance, Cardiorespiratory Profile, and Tether Force in Youth Trained Swimmers

**DOI:** 10.3390/life13091806

**Published:** 2023-08-24

**Authors:** Mário C. Espada, Cátia C. Ferreira, José M. Gamonales, Víctor Hernández-Beltrán, Danilo A. Massini, Anderson G. Macedo, Tiago A. F. Almeida, Eliane A. Castro, Dalton M. Pessôa Filho

**Affiliations:** 1 Instituto Politécnico de Setúbal, Escola Superior de Educação, 2914-504 Setúbal, Portugal; catia.ferreira@ese.ips.pt; 2 Life Quality Research Centre (CIEQV), Complexo Andaluz, Apartado, 2040-413 Rio Maior, Portugal; 3 Interdisciplinary Centre for the Study of Human Performance (CIPER), Faculdade de Motricidade Humana, Universidade de Lisboa, 1499-002 Lisbon, Portugal; 4 Research Group in Optimization of Training and Performance Sports (GOERD), Faculty of Sport Science, University of Extremadura, 10005 Cáceres, Spain; martingamonales@unex.es (J.M.G.); vhernandpw@alumnos.unex.es (V.H.-B.); 5 Facultad de Ciencias de la Salud, Universidad Francisco de Vitoria, 28223 Madrid, Spain; 6 Department of Physical Education, School of Sciences, São Paulo State University (UNESP), Bauru 17033-360, Brazil; dmassini@hotmail.com (D.A.M.); andersongmacedo@yahoo.com.br (A.G.M.); tiagofalmeida.w@gmail.com (T.A.F.A.); eliane.castro@unesp.br (E.A.C.); dalton.pessoa-filho@unesp.br (D.M.P.F.); 7 Postgraduate Program in Human Development and Technology, Biological Institute (IB), São Paulo State University (UNESP), Rio Claro 13500-000, Brazil; 8 Laboratory of Exercise Physiology Research Group (LFE—Research Group), Universidad Politécnica de Madrid (UPM), 28040 Madrid, Spain

**Keywords:** absorptiometry, gas exchange, force, performance, stationary, swimming

## Abstract

This study sought to analyze the relationship between regional body composition, swimming performance, and aerobic and force profile determined through tethered swimming in well-trained swimmers. Eleven male and five female swimmers were involved in the study and underwent the following evaluations: (1) body composition, assessed by the dual-energy X-ray absorptiometry method (DXA); (2) swimming performance, determined for 200, 400, 800, and 1.500 m front-crawl swimming; (3) a tethered swimming force test to determine maximum and mean force (F_max_ and F_mean_); and (4) an incremental tethered swimming test for the aerobic profile determination of the swimmers. Oxygen uptake (VO_2_) was directly measured by an automatic and portable system (K4b^2^ Cosmed, Italy). The fat-free mass (lean mass + bone mineral content, LM+BMC) in lower and upper limbs (UL_LM+BMC: 6.74 ± 1.57 kg and LL_LM+BMC: 20.15 ± 3.84 kg) positively correlated with all indexes of aerobic conditioning level, showing higher coefficients to the indexes representing the ability to perform at high aerobic intensities (VO_2max_: 49.2 ± 5.9 mL·kg^−1^·min^−1^ and respiratory compensation point (RCP): 43.8 ± 6.0 mL·kg^−1^·min^−1^), which attained 0.82 and 0.81 (with VO_2max_), 0.81 and 0.80 (with RCP). The S_200_ (1.48 ± 0.13 m·s^−1^) was significantly correlated to Trunk_LM+BMC (r = 0.74), UL_LM+BMC (r = 0.72), Total_LM+BMC (r = 0.71), and LL_LM+BMC (r = 0.64). This study highlights that regional body composition plays an important role in swimming, and body segment analysis should be considered instead of the total body. Tethered swimming may represent a useful method for force and aerobic assessment, aiming at training control and performance enhancement.

## 1. Introduction

Research in sports has been searching for methodologies that are less time-consuming and that, at the same time, use instruments that are not only at specific training facilities but that allow portability, which is particularly important in sports with a highly competitive density and that take place in environments that pose constraints to regular training control, as is the case with swimming. Despite the assumption that body composition plays an important role in sports [1], particularly in swimming, where body composition control was shown to be a valuable tool to optimize competitive performance through monitoring the efficiency of body adaptation to the training process [2], most research has focused on biomechanical and physiological evaluation [3,4,5,6,7,8], since these have been considered and assumed as performance indicators in this specific sport, which is quite demanding for athletes from a training point of view.

Swimming races are defined to integrate sprint distances (50–100 m), middle distances (200–400 m), and long distances (800–1500 m) [9], with more or less 86% of indoor swimming events being performed at maximal or supramaximal intensities [10]. Hence, the accuracy in identifying exercise intensity domains toward the optimization of daily training plays an important role in performance enhancement in swimming [11], by athletes with appropriate body composition and high physical capacity corresponding to the specificity of the aquatic effort [4]. In swimming, the assessment of specific strength is deemed a key factor when performing an evaluation of athletes. For this purpose, swimming tests in a tethered condition have been applied [12,13], since the ability to produce the force useful for propulsion is fundamental for competitive success [14,15] and previous research has revealed important associations between force evaluated in tethered apparatuses and free-swimming performance in sprint events [16,17,18].

Moreover, several studies have corroborated that tethered swimming tests (lasting 30 to 120 s) provide force values strongly related to short- and middle-distance swimming performance [18,19], as well as incremental tests in tethered swimming, are reliable in defining different swimming intensity zones for domain-specific training [13]. Nevertheless, the number of studies in which oxygen uptake (VO_2_) was directly measured in young swimmers is considered limited [20] regarding the influence that body composition variables can have on the swimmers’ performance [4], despite research pointing out that the maximal oxygen uptake (VO_2max_) measured during free swimming is not significantly different and highly correlated compared to determination throughout a tethered swimming test [21].

Body composition fat, bone, muscle, and water mass are very important in sports [22], namely when considering athletes’ health and performance [1]. In recent years, bioelectrical impedance analysis (BIA), especially direct segmental multifrequency methods, have been widely used in science and sports practice—along with other traditional body composition methods such as skinfold measurements and dual-energy X-ray absorptiometry—and have become a standard method for the determination of complete body structure according to the body segments [23,24,25]. Moreover, it was indicated in the past that the morphological characteristics of young athletes may influence swimming performance and vary by events [26]; namely, regional and whole-body lean mass (LM) influence short-term performance, anaerobic reserves, and fat-free mass for upper limbs (UL), and, consequently, exercise intensity at VO_2max_ (iVO_2max_), which will naturally influence swimming performance [27].

Previous scientific evidence underlined that for strength measurement, tethered swimming is the most specific in-water test [28] and can be used with the purpose of evaluating the cardiorespiratory capacity of a swimmer [29], associated with indications that the relationship between the body composition and performance of swimmers has been a source of unceasing interest among scientists, especially in young athletes [30]. To our best knowledge, no study has analyzed the relationship between regional body composition, force, and aerobic variables determined throughout tethered swimming and performance in different distance swimming trials in well-trained swimmers. Hence, this study aimed to evaluate the relationship between regional body composition, swimming performance, force, and aerobic profile determined throughout tethered swimming in well-trained swimmers. The current study hypothesized that regional fat-free mass plays a substantial influence on the variables of aerobic conditioning level, stroke force, and swimming performance and that fat-free mass in arms and legs will probably show a greater influence than other body regions.

## 2. Materials and Methods

### 2.1. Study Design

The swimmers performed a total of two visits for data collection, the first to the laboratory and the second to the water training facility, with an interval of 48 h. During the first visit, all athletes (when older of age) or legal guardians were required to provide written informed consent prior to data collection, and the associated risks and potential benefits of participation were explained. Afterward, subjects performed anthropometric and body composition evaluations, and later in the same day subjects were again familiarized with tethered swimming, something previously integrated into the routines of the athletes in training and research.

During the second visit, subjects performed the tethered swim tests to evaluate the force values that would be employed during the incremental test to determine aerobic variables. Free swimming performance in 200, 400, 800, and 1.500 m front-crawl swimming was evaluated on separate days in a training environment and the corresponding swimming speeds were determined, respectively, S_200_, S_400_, S_800_, and S_1.500_.

### 2.2. Participants

Sixteen swimmers, eleven males (18.0 ± 4.0 years of age; 1.80 ± 0.07 m of height; 71.8 ± 9.5 kg of body mass and 22.1 ± 2.9 kg/m^2^ of body mass index—BMI) and five females (16.8 ± 3.6 years of age; 1.66 ± 0.06 m of height; 61.1 ± 9.8 kg of body mass and 22.0 ± 2.1 kg/m^2^ of BMI), participated in this study. They were experienced at the national level, with the best 200 m front-crawl performance representing 554.0 ± 128.6 International Swimming Federation (FINA) points, and each accumulated a minimum of five years of competitive training with yearly regular participation in official swimming events and a weekly in-water training routine of six to seven training sessions of around 32 km, as well as two to three dry-land workouts.

Participants were instructed to: (1) avoid strenuous exercise 24 h prior to each moment of data collection and (2) arrive at sports facilities fully hydrated and rested. In order to mitigate the influence of circadian rhythms or variations in prior exercise, uniform environmental conditions were maintained throughout all tests, namely a specific time of day (±2 h), a water temperature of approximately 28 °C, relative humidity of around 50%, and all participants followed an identical pre-test warm-up protocol. The study was conducted considering the international ethical standards for sport and exercise science research [31] and the Declaration of Helsinki. It was also submitted and approved by the Ethical Committee of the São Paulo State University (UNESP) (registration nº CAEE: 02402512.7.0000.5398, and process nº 237.706).

### 2.3. Procedures

#### 2.3.1. Anthropometric and Morphological Evaluations

The dual-energy X-ray absorptiometry method with Discovery Wi device (Hologic Inc., Bedford, MA, USA) was used to evaluate regional and total body composition. The total mass (TM), fat mass (FM), and fat-free mass (lean mass + bone mineral content, LM+BMC) were obtained for the body regions (legs and arms on the right and left sides of the body and trunk) and for the whole body. Furthermore, TM, FM, and LM+BMC of the UL and lower limbs (LL) were obtained by adding the respective pairs of variables for each region.

The manufacturer’s recommendations were followed for equipment calibration and the entire procedure was operated by a knowledgeable technician. The evaluation procedures followed the suggestions of Nana et al. [32]: (a) the participants presented themselves with light clothes, without shoes, and without carrying any metallic objects or accessories next to the body; and (b) they remained lying down in dorsal decubitus, with 15 cm of distance between the feet, hands in a semi-pronation position and 3 cm from the trunk along the side of the trunk. The anatomical alignment obeyed the points specified by the program.

#### 2.3.2. In-Water Tests

**Figure 1:**
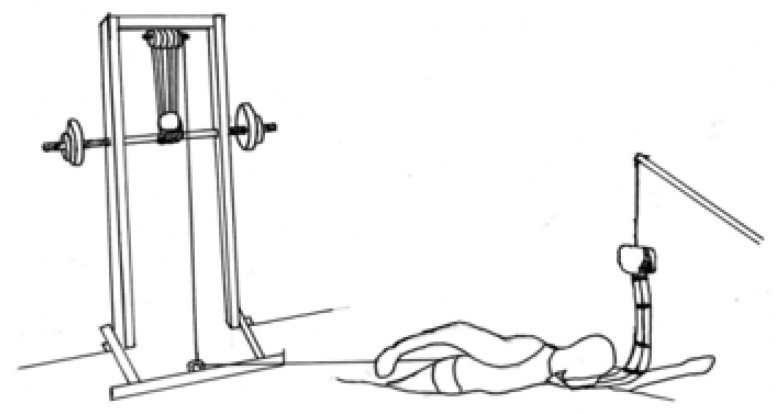
Schematic representation of tethered swimming procedure.

Tests were undertaken in a short-course swimming pool (25 m). An inelastic rope was used, with a 4905 N load cell attached to the swimmer’s hip in the all-out tethered swim test, associated with a custom-built weight-bearing pulley-rope system similar to a power rack but adapted for instantaneous weight-plate loading (≥0.4-kg increments) (Figure 1). The athletes swam in an all-out front crawl for 30 s with the averaged peaks of the wave frequency from the force-time signal defined as the trial’s mean force. The test was performed twice, assuming a 20 min rest, and the higher assessment for mean force (Fmean) was recorded. The load cell was calibrated for 100 Hz signal acquisition prior to each test and the signal was smoothed through the manufacturer’s software package (N2000PRO, Cefise, São Paulo, Brazil). Afterward, the difference between Fmean and the force that was required to maintain the swimmer’s body alignment prior to the initiation of the all-out swim (i.e., baseline force production; Fbase) was determined to derive ΔF.

**Figure 2:**
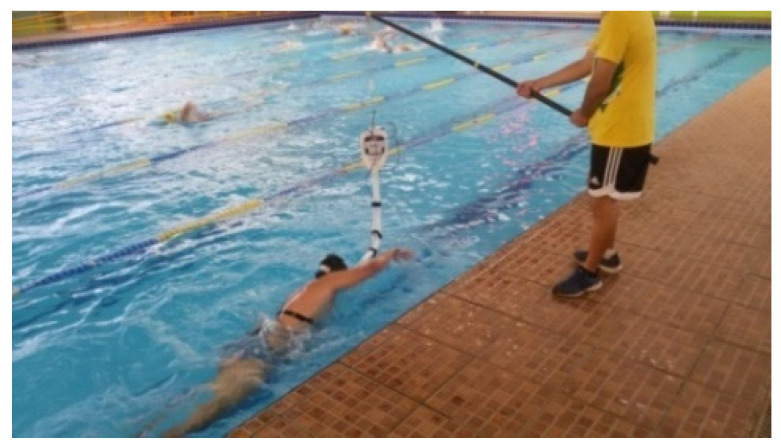
Image of data collection associated with the tethered swimming procedure.

With respect to the incremental test, the weight plates were loaded manually by the supervisors after receiving time signals from an associate. In all tests performed with this equipment, the attachment of the rope considering the indicated details allowed the leg kick to be unimpeded while providing a near-horizontal opposing force, which caused minimal alteration of the standard swimming posture (Figure 2). Load increments were applied during each stage and swimmers were instructed to perform at an adequate rate to prevent the rearward/forward displacement of their body position, and the stage length was 60 s. The initial stage was completed considering a load exceeding Fbase by 30% of ΔF, and from that point, each stage comprised a load increment of 5% of ΔF.

With the objective of swimmers to maintain a relatively fixed position (e.g., ±1 m from the desired position), visual reference points were implemented through two markers on the bottom of the pool, and the test was stopped when this criterion was longer possible. Breath-by-breath pulmonary gas-exchange data were collected with a portable metabolic unit (CPET K4b^2^; Cosmed, Rome, Italy) with swimmers breathing through a snorkel apparatus (new AquaTrainer) previously validated for swimming [33].

Previous to each test, the manufacturer’s recommendations were followed regarding unit calibration. After this procedure and before the swimmer’s attachment, all the athletes rested for 10 min on the border of the pool to establish baseline variables. The VO_2_ data measured during the baseline and swimming periods were averaged over consecutive 9 s periods after being smoothed by the collection unit’s software. The VO_2_max was defined as the highest three-point rolling average of consecutive 9 s VO_2_ values recorded before the limit of tolerance. The final three-point rolling average for each completed 60 s stage was considered to determine the VO_2_/load slope through linear regression. When VO_2_ failed to increase by a visible amount for ≥2 stages immediately preceding the limit of tolerance, a VO_2_ plateau was considered, and the datum from that stage was removed from the fit.

The GET and RCP identifications resulted from the consensus of a panel of independent and experienced reviewers from a cluster of measurements. Considering GET, these included (1) the first disproportionate increase in the rate of carbon dioxide production (VCO_2_) from the visual inspection of individual plots of VCO_2_ vs. VO_2_, an increase in the expired rate of ventilation VE/VO_2_ with no increase in VE/VCO_2_; and (2) an increase in end-tidal O_2_ tension with no fall in end-tidal CO_2_ tension. Regarding RCP, the criteria included (1) the first disproportionate increase in VE in relation to VCO_2_; and (2) a fall in end-tidal CO_2_ tension.

Free swimming performance was evaluated in a training environment considering the performance in 200, 400, 800, and 1.500 front-crawl swimming, and the correspondent swimming speed was determined (S_200_, S_400_, S_800,_ and S_1.500_).

### 2.4. Statistical Analysis

All data were initially computed as means and standard deviations (M ± SD) in Microsoft Excel™ and all additional analyses were computed in Statistical Package for Social Science v27.0 (SPSS Inc., Armonk, NY, USA). The normality of data was first checked by the Shapiro–Wilk test. Linear regression models were computed. Trendline equation and determination coefficients (R^2^) were calculated and categorized as <0.04 (trivial), 0.04–0.24 (small), 0.25–0.63 (medium), and >0.64 (strong) [34].

Pearson’s r correlation coefficients were calculated, with the absolute value demarcated as follows [35]: negligible correlation (r < 30), weak correlation (r = 0.30–0.50), moderate correlation (r = 0.50–0.70), strong correlation (r = 0.70–0.90), and very strong correlation (r > 90). The sample power (SP) was determined (GPower, v.3.1.9, University of Kiel, Kiel, Germany) from post-data results of Pearson’s coefficient (r), actual N sample (N = 16), and specifying a security level at α = 0.05 [36]. Statistical significance was accepted at *p* ≤ 0.05.

## 3. Results

Participants’ swimming performance was 1.48 ± 0.13 m·s^−1^, 1.34 ± 0.10 m·s^−1^, 1.25 ± 0.12 m·s^−1^, and 1.25 ± 0·09 m s^−1^, respectively, for S_200_, S_400_, S_800_, and S_1.500_. Descriptive statistics displaying the M ± SD of physiological responses during incremental stepwise tethered swimming tests are presented in Table 1.

The RCP corresponded to 89.99% and GET to 64.70% of VO_2max_. Moreover, iRCP was 87.55% and iGET 61.50% of iVO_2max_. Table 2 presents the regional body composition variables determined through DXA, F_max_, and F_mean_ measured in the tethered swimming test.

**Figure 3:**
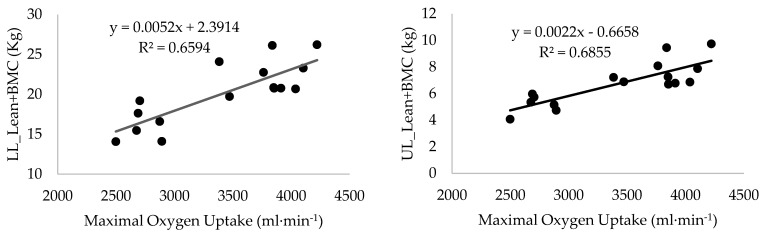
Linear regression of maximal oxygen uptake on lower and upper limb lean mass + bone mineral content. SP = 0.99 (**left** and **right** panels).

Table 3 shows the correlations between all regional body compositions and aerobic and force variables determined in tethered swimming, and Figure 3 depicts the linear regression of VO_2max_ on UL and LL LM+BMC. The correlations showed moderate to high coefficients between the regional fat-free mass (UL_LM+BMC, Trunk_LM+BMC, and LL_LM+BMC) to the physiological responses and workload corresponding to both thresholds (GET and RCP), maximal aerobic intensity (VO_2max_), as well as to the ability to generate force while stroking.

The S_200_ was significantly correlated to Trunk_LM+BMC (r = 0.74, *p* < 0.01, SP = 0.97), UL_LM+BMC (r = 0.72, *p* < 0.01, SP = 0.96), Total_LM+BMC (r = 0.71, *p* < 0.01, SP = 0.95), and LL_LM+BMC (r = 0.64, *p* < 0.01, SP = 0.88). Moreover, S_200_ was also significantly correlated to Total_FM (r = −0.56, *p* < 0.05, SP = 0.77), UL_ FM (r = −0.55, *p* < 0.05, SP = 0.75), and both Trunk_FM and LL_FM (r = −0.54, *p* < 0.05, SP = 0.73). With respect to TM, only specifically in UL, a correlation was observed with S_200_ (r = 0.56, *p* < 0.05, SP = 0.77). S_400_, S_800,_ and S_1_._500_ were not correlated with regional body composition variables. Figure 3 presents the linear regressions between VO_2max_ and both LL and UL fat-free mass.

Considering aerobic variables determined in tethered swimming, S_1.500_ was significantly correlated to VO_2max_ and iVO_2max_ (r = 0.52, *p* < 0.05, SP = 0.70) and _i_GET (r = 0.51, *p* < 0.05, SP = 0.68). Moreover, S_800_ was significantly correlated to VO_2max_ (r = 0.76, *p* < 0.05, SP = 0.98), iVO_2max_ (r = 0.56, *p* < 0.05, SP = 0.77), RCP (r = 0.53, *p* < 0.01, SP = 0.71), iRCP (r = 0.54, *p* < 0.01, SP = 0.73), and iGET (r = 0.74, *p* < 0.01, SP = 0.97).

**Figure 4:**
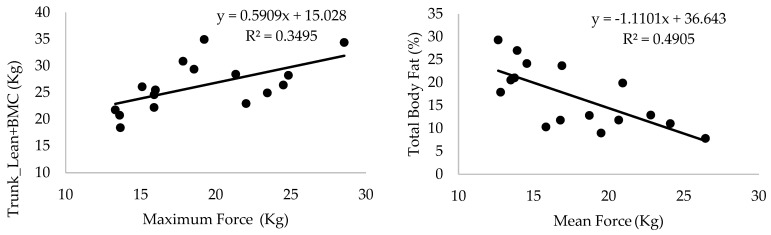
Linear regression of maximum and mean force on trunk lean mass + bone mineral content and total fat mass (%). SP = 0.85 and SP = 0.94 (**left** and **right** panels, respectively).

Likewise, S_200_ was significantly correlated to VO_2max_ (r = 0.55, *p* < 0.05, SP = 0.75), iVO_2max_ (r = 0.70, *p* < 0.01, SP = 0.94), RCP (r = 0.52, *p* < 0.05, SP = 0.70), and iRCP (r = 0.55, *p* < 0.05, SP = 0.63). No correlations were observed between S_400_ and regional body composition variables. Figure 4 shows the linear regression of F_max_ and F_mean_ on regional body composition variables.

Both F_max_ and F_mean_ were negatively correlated to FM in LL, UL, and trunk, and swimming performance only correlated to S_200_ (respectively, r = 0.55, SP = 0.75 and r = 0.51, SP = 0.68, in both cases *p* < 0.05), despite multiple positive correlations with regional body composition variables.

## 4. Discussion

The purpose of this research was to analyze the relationship between regional body composition, swimming performance, and aerobic and force profiles determined throughout tethered swimming in well-trained swimmers. The main findings were: (1) only S_200_ was associated with correlations with body composition variables, namely positive correlations to regional LM+BMC and TM and negative correlations to FM); (2) force variables (F_max_ and F_mean_) were positively correlated to LM+BMC and TM and negatively correlated to FM; (3) positive correlations were observed only between S_200_ and both F_max_ and F_mean_; (4) all aerobic variables (VO_2max_, RCP and GET) and the corresponding exercise intensities (iVO_2max_, iRCP, and iGET) determined through tethered swimming were correlated to both F_max_ and F_mean_; and (5) swimming performance, namely S_1.500_, S_800,_ and S_400_, was positively correlated to aerobic variables (VO_2max_, RCP and GET) and the respective exercise intensities determined through tethered swimming.

Previous research found a BMI value of 22.78 in high school and university swimmers [37]. Also, Gagnon et al. [38], analyzing the 2012 Olympic Games, found that BMI was not significantly associated with event distance in men’s or women’s swimming (from 50 m to 10 km), and observed a mean BMI ~ 23 in both male and female swimmers, concluding that power and VO_2max_ are the main causes of differences in race performance in elite athletes in these swimming events. Our results of BMI confirm that swimmers regularly engaged in training present BMI values within the healthy range (18.5 to 24.9), but on the other hand, it reveals that this indicator by itself is not the best indicator or performance predictor.

The lower FM most likely results in lower body shape drag (frontal area) and skin friction drag, while, simultaneously, body composition contractile potential provides a better propulsion force potential for faster swimming [39]. Additionally, a larger body and increased surface area will increase drag, associated with a decreased racing speed for a given amount of mechanical power [40]. Earlier research also revealed that there is significant evidence that fat reduction contributes to muscular and cardio-respiratory endurance as well as to the development of speed and agility [22,41]. The FM and LM both seem to contribute to swimmers’ performance [42,43]. Previously, Avlonitou et al. [44] studied the effects of competitive swimming on body composition, verifying that the bone density and LM in the LLs were not affected by swim training, despite a decrease in FM observed. Although, during a competitive swim season, a significant increase in LM and a decrease in FM has been associated with the part of the season when training is intense [45].

This present research found that body segments (LL, UL, and truck) and corresponding tissue content (TM, FM, and LM+BMC) reinforce the importance of body composition evaluation in swimming, showing that detailed analysis assessed by a DXA methodology can provide useful insights into the relative influence of regional body composition for swimming performance. Strength and power are highly connected with muscle size [22,39]; consequently, thus, an increase in muscle or LM enables the athletes to produce more muscle force during specific movement efforts, which improves speed, quickness, acceleration, and agility [46,47]. Earlier, Nevill et al. [48], showed that LM was the singularly most important whole-body characteristic associated with front-crawl swim speeds. The role of UL muscle power is even more essential since 85–90% of the propulsive power derives from the arms, and swimmers primarily use their arms to generate forward thrust [49].

In our study, the positive correlations between LM+BMC and aerobic variables (VO_2max_, RCP and GET) were always higher when compared to TM and FM, highlighting that this specific body composition variable plays an important role in swimmers’ capacitation and may contribute to performance enhancement. Furthermore, regional LM+BMC was the only body composition variable with positive correlations to F_max_ and F_mean_, with higher values in UL, followed by LL and trunk. This evidence is of particular importance not only for training control and performance enhancement but also for injury prevention, since the majority of competitive swimmers devote a large part of daily time to in-water and land workouts and consequently are extremely exposed to overuse injuries [50].

Recently, Sokołowski et al. [51] found moderate to high partial correlations between particular periods of seconds in the 1 min VO_2_, 31–60, 41–60, and 51–60, and F_max_ and F_mean_, noting a relationship between 41–60 and 51–60 VO_2_ and the overall performance in 200 m front crawl (r = 0.64, *p* ≤ 0.01). These authors noticed a significant positive correlation between all indices and 200 m front-crawl speed (0.32 ≤ r ≤ 0.41, *p* ≤ 0.01). Other studies have reported similar findings. Santos et al. [18] verified a positive correlation (r = 0.61, *p* < 0.001) between the F_max_ of the 2 min tethered swimming test and the swimming speed of a 200 m front crawl, while another study showed a very strong relationship between F_mean_ and F_max_ and 200 m front-crawl swimming speed (r = 0.94 and r = 0.93, respectively, *p* < 0.01) [19].

In the present study, both F_max_ and F_mean_ were only correlated to S_200_ (respectively, r = 0.55 and r = 0.51, in both cases *p* < 0.05), despite multiple positive correlations with regional body composition variables and aerobic variables (VO_2max_, RCP, and GET) and the respective swimming intensities determined throughout tethered swimming (iVO_2max_, iRCP, and iGET), which is particularly important because 30–60 s of maximum effort could be enough to reach up to 90% of athletes’ VO_2max_ [52]. Moreover, our results also revealed that S_1.500_ was significantly correlated to VO_2max_, iVO_2ma_, and _i_GET. The S_800_ was significantly correlated to VO_2max_, iVO_2max_, RCP, iRCP, and iGET. Additionally, S_200_ was significantly correlated to VO_2max_, iVO_2max_, RCP, and iRCP. No correlations were observed between S_400_ and aerobic variables or regional body composition variables, such as in the case of S_800_ and S_1.500_. Nevertheless, S_200_ was significantly correlated to total, trunk, LL, and UL fat-free mass and negatively correlated to total, trunk, LL, and UL fat mass, in all cases with different values regarding body segments.

Coaches should acknowledge the importance of body composition analysis as a valuable tool in understanding swimmers’ physiological indexes and swimming performance. By assessing regional and total body fat-free mass, coaches can gain support for the athletes’ metabolic insights and make informed training decisions. For young swimmers, fat-free mass in the trunk, legs, and arms can influence the enhancement of both aerobic physiological indices through training and the capacity to generate strength, which, in turn, directly affects the increase in speed during middle-distance swimming events, particularly in the 200 m. Therefore, coaches should consider targeting and developing fat-free mass in specific body regions to optimize performance outcomes and enhance swimmers’ abilities in medium-distance events by considering the relationship with maximum and submaximal cardiorespiratory fitness.

It is important to acknowledge some limitations associated with this study. Firstly, we did not consider the swimmers´ maturation or their race specialty. The potential differences between swimming performance in short and long-course swimming pools were also not evaluated, which makes it impossible to generalize our results to the whole swimming community. Also, the smaller sample of female swimmers did not allow a comparison between sexes, and the results can be influenced by the mixed sample regarding the role of sex-specific lean mass and distribution of both muscular strength (which was not assessed in the current study) and tethered swimming force, despite muscle force improvement and hypertrophy with resistance training having been reported not to influence swimming velocity directly [53]. Future research should ponder different swimmers’ age categories, sex, and level, and the comparison between swimming performance in short and long-course swimming pools. The possible measurement and analysis of VO_2_ kinetics and blood lactate could also provide physiological insight into fatigue mechanics, which can also, in the future, be compared to biomechanical variables in tethered swimming.

## 5. Conclusions

This study confirms the hypothesis that regional fat-free mass is associated with physiological indices and swimming performance at intensities in the heavy to severe domain of exercise, as well as with the ability to apply force during the swimming stroke. In addition to these results, regional and body fat-free mass have an influence on swimming performance in 200 m. Therefore, it can be assumed that, among young swimmers, fat-free mass (in the trunk, legs, and arms) is a factor that contributes both to the increase in aerobic physiological indices with training, as well as to the increase in the capacity of produced strength, thus directly influencing the increase in speed in medium-distance swimming events (200 m) or indirectly by increasing the physiological indices (and performance capacity) corresponding to maximum and submaximal cardiorespiratory fitness. Future studies will need to answer whether strength training protocols in and out of the water, which aim to increase regional and body muscle mass, will be able to observe increases in aerobic fitness, as well as performance at different swimming distances.

## Figures and Tables

**Table 1:** Aerobic variables determined during the incremental tethered swimming test.

Variables	M	SD
Absolute VO_2max_ (mL·min^−1^)	3.423.0	601.8
Relative VO_2max_ (mL·kg·min^−1^)	49.2	5.9
iVO_2max_ (kg)	8.9	1.6
Absolute RCP (mL·min^−1^)	3.046.2	588.7
Relative RCP (mL·kg·min^−1^)	43.8	6.0
iRCP (kg)	7.8	1.4
Absolute GET (mL·min^−1^)	2.214.8	455.0
Relative GET (mL·kg·min^−1^)	32.0	6.3
iGET (kg)	5.5	1.3

M, mean; SD, standard deviation; VO_2max_, maximal oxygen uptake; RCP, respiratory compensation point; GET, gas exchange threshold; iVO_2max,_ intensity at maximal oxygen uptake; iRCP, intensity at respiratory compensation point; iGET, intensity at gas exchange threshold.

**Table 2:** Regional body composition variables determined through the dual-energy X-ray absorptiometry method and maximum and mean forces measured during the tethered swimming test.

Variables	M	SD
F_max_ (kg)	19.0	4.7
F_mean_ (kg)	17.7	4.3
Total_LM+BMC (kg)	56.8	10.1
TM (kg)	68.4	10.6
Total FM (%)	16.9	6.8
UL_LM+BMC (kg)	6.7	1.6
UL_TM (kg)	8.0	1.5
UL_FM (%)	16.0	8.6
Trunk_LM+BMC (kg)	26.2	4.6
Trunk_TM (kg)	30.8	5.1
Trunk_FM (%)	14.8	6.8
LL_LM+BMC (kg)	20.1	3.8
LL_TM (kg)	25.1	4.0
LL_FM (%)	19.6	8.1

M, mean; SD, standard deviation; F_max_, maximum force; F_mean_, mean force; TM, total mass_;_ Total FM, total fat mass, UL_LM+BMC, upper limb lean mass + bone mass content; Trunk_LM+BMC, trunk lean mass + bone mass content; LL_LM+BMC, lower limb lean mass + bone mass content.

**Table 3:** Correlations between body composition variables and aerobic and force variables determined in tethered swimming.

	VO_2max_	iVO_2max_	RCP	iRCP	GET	iGET	F_max_	F_mean_
UL_LM+BMC	0.83 **	0.80 **	0.81 **	0.74 **	0.76 **	0.68 **	0.70 **	0.68 **
UL_TM	0.80 **	0.72 **	0.77 **	0.69 **	0.56 *	0.65 **	-	-
UL_FM	-	-	0.99 **	-	−0.73 **	−0.65 *	−0.68 **	−0.72 **
Trunk_LM+BMC	0.78 **	0.76 **	0.76 **	0.65 **	0.60 *	0.61 *	0.59 *	0.58 *
Trunk_TM	0.69 **	0.57 *	0.64 **	0.55 *	-	0.52 *	-	-
Trunk_FM	-	-	-	-	-	-	−0.61 *	−0.65 **
LL_LM+BMC	0.81 **	0.72 **	0.80 **	0.68 **	0.67 **	0.62 **	0.63 **	0.62 *
LL_TM	0.69 **	0.54 **	0.64 **	0.54 **	0.67 **	0.62 **	-	-
LL_FM	-	-	-	-	−0.67 **	−0.63 **	−0.67 **	−0.71 **

M, mean; SD, standard deviation; VO_2max_, maximal oxygen uptake; RCP, respiratory compensation point; GET, gas exchange threshold; iVO_2max,_ intensity at maximal oxygen uptake; iRCP, intensity at respiratory compensation point; iGET, intensity at gas exchange threshold. F_max_, maximum force; F_mean_, mean force; UL_LM+BMC, upper limb lean mass + bone mass content; Trunk_LM+BMC, trunk lean mass + bone mass content; LL_LM+BMC, lower limb lean mass + bone mass content, TM, total mass; FM, fat mass. * (*p* < 0.05), ** (*p* < 0.01).

## Data Availability

The data that support the findings of this study are available from the first and last authors (mario.espada@ese.ips.pt and dalton.pessoa-filho@unesp.br).
